# Role of Gut Microecology in the Pathogenesis of Drug-Induced Liver Injury and Emerging Therapeutic Strategies

**DOI:** 10.3390/molecules29112663

**Published:** 2024-06-04

**Authors:** Yuqiao Huang, Yu Zhang, Kaireng Wu, Xinxin Tan, Tian Lan, Guixiang Wang

**Affiliations:** 1 School of Pharmacy, Guangdong Pharmaceutical University, Guangzhou 510006, China; 2 Department of Pharmacology, College of Pharmacy, Harbin Medical University, Harbin 150086, China

**Keywords:** drug-induced liver injury, gut microecology, gut microbiota, molecular mechanisms

## Abstract

Drug-induced liver injury (DILI) is a common clinical pharmacogenic disease. In the United States and Europe, DILI is the most common cause of acute liver failure. Drugs can cause hepatic damage either directly through inherent hepatotoxic properties or indirectly by inducing oxidative stress, immune responses, and inflammatory processes. These pathways can culminate in hepatocyte necrosis. The role of the gut microecology in human health and diseases is well recognized. Recent studies have revealed that the imbalance in the gut microecology is closely related to the occurrence and development of DILI. The gut microecology plays an important role in liver injury caused by different drugs. Recent research has revealed significant changes in the composition, relative abundance, and distribution of gut microbiota in both patients and animal models with DILI. Imbalance in the gut microecology causes intestinal barrier destruction and microorganism translocation; the alteration in microbial metabolites may initiate or aggravate DILI, and regulation and control of intestinal microbiota can effectively mitigate drug-induced liver injury. In this paper, we provide an overview on the present knowledge of the mechanisms by which DILI occurs, the common drugs that cause DILI, the gut microbiota and gut barrier composition, and the effects of the gut microbiota and gut barrier on DILI, emphasizing the contribution of the gut microecology to DILI.

## 1. Introduction

The hepatotoxicity associated with exogenous substances, such as pharmaceuticals, natural compounds, and chemical agents, constitutes a significant etiology of liver damage [1]. Drug-induced liver injury (DILI) is one of the most common clinical adverse drug reactions and refers to hepatotoxicity induced by various chemical drugs, herbal medicines, natural drugs, biologics, nutraceuticals, and dietary supplements and their metabolites [2]. The development of DILI is accompanied by structural changes in the gut microecology, and regulation of the gut microbiota can effectively mitigate DILI. The human body has trillions of microbial cells and their synergy is considered important for human health. Microbial cells have the highest density in the gut, and together they form a complex microbiome called the gut microbiome [3]. The gut barrier is the sum of the structures and functions of the intestine that prevent harmful substances such as bacteria and endotoxins in the gut lumen from crossing the intestinal mucosa into other tissues, organs, and blood circulation; the gut microbiota and the gut barrier are interconnected to achieve a complex network of interactions. Modulation of gut microbe–gut barrier interactions is increasingly recognized as a target for new therapeutic strategies for several intestinal and extraintestinal diseases [4]. Drugs can affect the composition and function of the gut microbiota. The gut microbiota can directly participate in drug metabolism affecting drug efficacy and toxicity. It can also interact with the immune/metabolic system, indirectly affecting drug response and bioavailability [5]. There is evidence that the gut microbiota and gut barrier play an important role in the development of DILI [6]. The gut and liver are anatomically linked by the biliary tract, the products of portal system and circulation from the immune system and the gut microbiota forming the “gut-liver axis”. Pathogen-associated molecular patterns (PAMPs) from the intestine reach the liver through the portal system and enter the body circulation through the mesenteric lymph nodes, where they bind to Toll-like receptors (TLRs) and lead to the activation of inflammatory pathways [7,8,9]. A comprehensive understanding of DILI and the gut is required to understand how the gut affects drug-related liver injury. We review the mechanisms of DILI, common drugs that cause DILI, the gut microbiota, the composition of the gut barrier, and the mechanisms by which different drugs affect DILI by acting on gut microbiota, expecting to find the interconnection between them and provide new therapeutic strategies for the treatment of DILI.

## 2. Drug-Induced Liver Injury (DILI)

DILI is a significant drug safety issue and one of the main reasons for withdrawing drugs shortly after launch [10]. Clinical symptoms may include cholestasis, hepatic steatosis, and hepatic fibrosis, which can lead to acute hepatic failure and even death in severe causes [11]. The liver toxicity website liverTox, sponsored by the National Institutes of Health, has described more than 1200 drugs that can cause liver damage [12]. Nonsteroidal anti-inflammatory drugs, anti-infective drugs, herbal medicines, and dietary supplements are common causes of DILI in the developed countries in Europe and the United States. Among them, acetaminophen (APAP) is the most important cause of acute liver failure (ALF) [10,13]. In mainland China, the annual incidence rate in the general population was estimated to be 23.80 per 100,000 people, higher than that reported in Western countries, with traditional Chinese medicine or herbal and dietary supplements and anti-tuberculosis drugs being the main causes of DILI, accounting for 26.81% and 21.99%, respectively [14]. After consulting the relevant literature, we systematically classified drugs commonly associated with DILI and synthesized a summary of their underlying mechanisms (Table 1).

**Figure 1:**
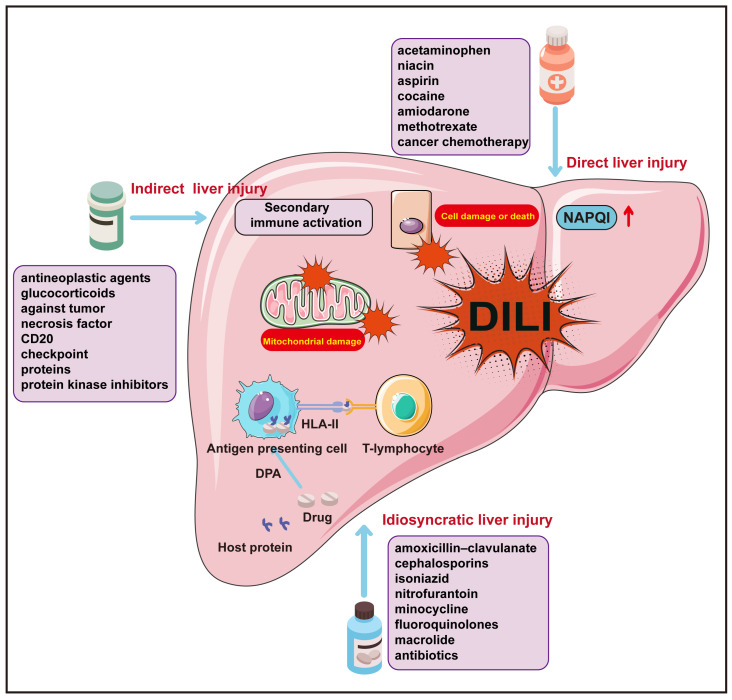
The molecular mechanisms of DILI. Direct hepatotoxicity is caused by agents that are intrinsically toxic to the liver. The incidence of idiosyncratic liver injury is predominantly related to the unique physiological constitution of the patient. Indirect hepatotoxicity of drugs refers to liver toxicity that is secondary to the pharmacological effects of a medication, rather than being an inherent or specific hepatotoxic quality of the drug itself. The arrows down indicate downregulation.

Current studies have shown that the occurrence of DILI is the result of a combination of multiple factors [15,16]; the mechanisms of occurrence involve direct, idiosyncratic, and indirect liver injury, each presenting with unique clinical characteristics. Beyond these categories, certain pharmaceuticals also exhibit oncogenic or carcinogenic potential, such as the correlation between androgens and oral contraceptives with hepatic adenomas, and aristolochic acid with hepatocellular carcinoma [17,18]. These particular cases of DILI are not discussed herein. Direct liver injury from a drug stems from the intrinsic damage to liver cells caused by the drug itself or its metabolic byproducts, with the most common example being the hepatotoxicity of acetaminophen, whose metabolite, N-acetyl-p-benzoquinone imine (NAPQI), is the toxic agent. Idiosyncratic liver injury is unforeseeable, not directly dose dependent, and not easily reproducible in animal models [19]. Immunologically mediated idiosyncratic hepatotoxicity arises when drugs or their metabolic byproducts couple with host proteins to form drug–protein adducts (DPAs) that furnish suitable antigenic epitopes, with the host furnishing an HLA-restricted immunologic response pathway [20]. Indirect liver injury constitutes a form of hepatic impairment incited by drug action, distinct from intrinsic hepatotoxicity or specific reactions linked to the drug in question [21]. This condition is often principally associated with the extensive secondary immune activation following the administration of immune checkpoint inhibitors (ICIs) [22,23,24]. Regardless of whether it is direct, idiosyncratic, or indirect liver injury, the ultimate outcome is mitochondrial damage and dysfunction in hepatocytes, precipitating varying degrees and extents of target cell injury and death (Figure 1) (Table 2) [25,26].

Recent research has noted diminished diversity within the gut microbiota of both DILI patients and animal models [27,28,29,30,31,32,33,34,35]. This includes an observed proliferation of potentially pathogenic bacteria, coupled with a decline in the relative abundance of inherently beneficial microbial groups (Table 3).

**Table 1:** Common types of drugs that cause DILI and common drugs [36,37,38,39].

Drug Type	Common Drugs
Nonsteroidal Anti-inflammatory	APAP, Celecoxib, Diclofenac, Nimesulide
Herbal And Dietary Supplements	Polygonum Multiflorum Thunb, Cinobufacini, Xianlinggubao, Guizhi Fuling Capsule, Tripterygium Wilfordii
Anti-Tuberculosis	Isoniazid, Rifampin, Pyrazinamide, Thiacetazone, Protionamide
Anti-Fungal Drugs	Ketoconazole, Fluconazole, Itraconazole, Voriconazole
Antibiotic	Amoxycillin/Clavulanic Acid, Vancomycin, Pefloxacin, Enoxacin, Ofloxacin, Ciprofloxacin, Roxithromycin, Azithromycin, Clarithromycin, Vancomycin, Norvancomycin, Lincomycin, Clindamycin
Central Nervous System Drugs	Chlorpromazine, Trifluoperazine, Risperidone, Phenobarbital, Valproate, Carbamazepine
Iron	Polysaccharide Iron Complex, Ferrous Succinate, Ferrous Fumarate, Ferrous Gluconate

**Table 2:** The molecular mechanisms of DILI [40,41,42,43,44,45].

	Direct Liver Injury	Idiosyncratic Liver Injury	Indirect Liver Injury
Specific Mechanisms	The direct (inherent) liver injury of drugs and their active metabolites, along with the body’s intrinsic pathophysiological damage response.	Pharmacometabolic dysfunctions associated with human genetic polymorphism, or drug–host protein conjugate specificity and human leukocyte antigen (HLA)-restricted acquired immune responses.	The biological activity secondary to pharmaceuticals or their active metabolites often exert hepatotoxic effects indirectly through modulating the immune system.
Clinical Manifestations	Elevated liver enzymes, acute hepatic necrosis, hepatic sinusoidal obstruction syndrome, acute fatty liver, and nodular regeneration, among others.	Acute hepatocellular injury, cholestatic hepatitis, mixed hepatitis, simple cholestasis, and chronic hepatitis, among others.	Acute hepatitis, immune-mediated hepatitis, steatohepatitis, and chronic hepatitis, among others.
Common Drugs	Acetaminophen, aspirin, methotrexate, and other antineoplastic chemotherapy agents, highly effective antiretroviral drugs, synthetic anabolic steroids, statins, cyclosporine, heparin, valproic acid, niacin, butyrate, cocaine, amiodarone, tacrolimus, and so forth.	Amoxicillin/clavulanate, flucloxacillin, cephalosporins, macrolides, nitrofurantoin, minocycline, allopurinol, propylthiouracil, diclofenac, leflunomide, thalidomide, lapatinib, pazopanib, flutamide, and so forth.	Immunotherapy checkpoint inhibitors, antitumor necrosis factor monoclonal antibodies, anti-CD20 monoclonal antibodies, protein kinase inhibitors, corticosteroids, select anti-neoplastic agents, and drugs related to energy metabolism and interfering substances.

**Table 3:** Microbiome changes in DILI.

Study Subjects	Drug Involved	Microbial Composition Shifts Compared to Normal Controls		Source
		Increased	Decreased	
Mice	Methotrexate (MTX)	*Firmicutes*; *Actinobacteriota*; *Proteobacteria*; *Blautia*; *Ruminococcu_torques_group*; *Staphylococcus*; *Enterorhabdus*; *Enterococcus*	*Bacteroidota*; *Verrucomicrobiota*; *Lactobacillus*; *Allobaculum*; *norank_f_Muribaculaceae*; *Dubosiella*; *Ruminococcus*	[27]
DILI patient; Healthy controls	-	*Bacteroides*; *Alistipes*	*Acetobacteroides*; *Blautia*; *Caloramator*; *Coprococcus*; *Flavobacterium*; *Lachnospira*; *Natronincola*; *Oscillospira*; *Pseudobutyrivibrio*; *Shuttleworthia*	[28]
DILI patient; Healthy controls	-	*Bacteroidota*; *Firmicutes*; *Fusobacteriota*; *Acidobacteriota*; *Streptococcus*; *Faecalibacterium*; *Bacteroides*; *Klebsiella*; *Enterococcus*; *Veillonella*	*Proteobacteria*; *Verrucomicrobiota*; *Desulfobacterota*; *Streptococcus*; *Faecalibacterium*; *Bacteroides*; *Klebsiella*; *Blautia*; *Ralstonia*; *Dialister*	[29]
Mice	Perfluoro-octanoic acid	*Parabacteroides*	*Dehalobacterium*; *Bacteroides*; *Lactobacillus*; *Bifidobacterium*	[30]
Mice	Acetaminophen (APAP)	*Akkermansia muciniphila*; *Verrucomicrobiales*; *Verrucomicrobiaceae*	*Firmicutes*; *Clostridia*; *Clostridiales*; *Lachnospiraceae*	[31]
Rats	Bisphenol A (BPA)	*Prevotellaceae_NK3B31_group*; *Firmicutes*	*Prevotella_9*; *Bacteroidetes*; *Ruminococcaceae_UC-G014*	[32]
DILI patient; Healthy controls	Antithyroid drugs (ATDs)	*Eubacteriumrectale*; *Romboutsia* *Dorea*	*Faecalibacterium*; *Clostridium_sensu_stricto_1*; *Bacteroides*	[33]
Rats	Oral iron	*Defluviitaleaceae UCG-011*; *RuminococcaceaeUCG-014* *Coprococcus 1*	*Lachnospiraceae FCS020*; *genus Allobaculum*	[34]
Rats	Tacrine	*Bacteroides*	*Lactobacillus*	[35]

## 3. Gut Microbiota

**Figure 2:**
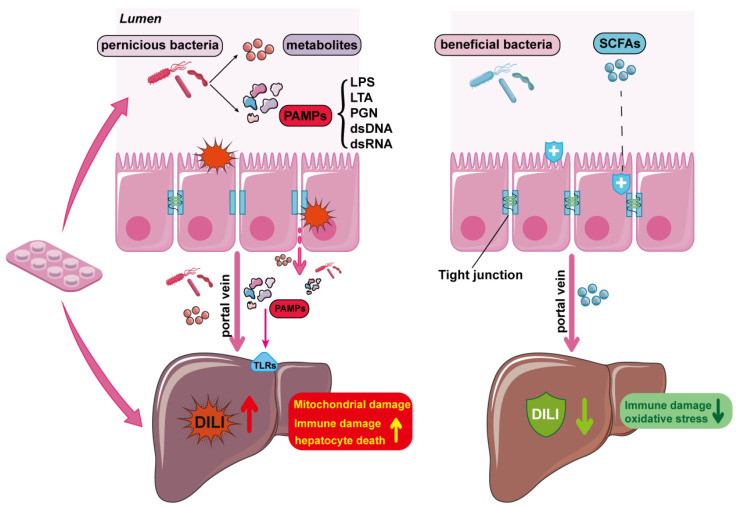
Intestinal microbiome influences DILI. When the intestinal barrier is compromised, translocated bacteria and microbial toxins can gain axis to distant sites. Bacteria, their metabolites, and PAMPs can enter the portal circulation and access the liver, exacerbating DILI. TLRs are multiprotein complexes that recognize PAMPs such as bacterial peptidoglycans (PGN) or lipopolysaccharide (LPS), double-stranded DNA and RNA (dsDNA, dsRNA), and lipoteichoic acid (LTA). SCFAs, the metabolites produced by the gut microbiota, play a critical role in regulating the balance between the function and morphology of the mucosal barrier, regulating the proliferation and differentiation of mucosal cells, protecting the integrity and permeability of the mucosal barrier, and maintaining the stability of tight junctions. Portal circulation allows intestinal-derived SCFAs to flow to the liver, reducing hepatic inflammatory injury and oxidative stress. The arrows pointing up indicate upregulation and the pointing down indicate downregulation.

The gut microbiota is a diverse ecosystem of bacteria, protozoa, archaea, fungi, and viruses and is known to establish complex trophic relationships with itself and its human host, ranging from symbiotic to parasitic [46]. Additionally, it is known as the “new virtual metabolic organ” [47]. The gut microbiota has been implicated in the pathogenesis of several diseases, such as infectious and noninfectious chronic liver disease, autism, cancer, depression, inflammatory bowel disease, irritable bowel syndrome, type 2 diabetes, colorectal cancer, atherosclerosis, obesity, and chronic kidney disease [48,49,50,51,52]. Among these systemic organs, the liver is uniquely positioned to receive signals from the gut microbiome. The liver receives most of the blood from the gut through the portal vein and is, therefore, most exposed to potential bacterial products or metabolites such as lipopolysaccharides, peptidoglycans, short-chain fatty acids (SCFAs), and bile acids. Bacterial products or metabolites can activate Kupffer cells, neutrophils, hepatocytes, sinusoidal endothelial cells, and stellate cells, promoting the release of inflammatory mediators, such as tumor necrosis factor alpha and interleukin 6, leading to liver injury and diseases [47]. The gut microbiota can directly influence the response of an individual to a specific drug by altering the structure of the drug through enzymatic reactions, changing its bioavailability, bioactivity, or toxicity, a phenomenon now known as pharmacomicrobiomics [53]. Furthermore, the gut microbiota can regulate the host expression of genes involved in various metabolic pathways, including nuclear receptor regulation, phase I and II enzymes, and transporters [54]. Moreover, the gut microbiota can produce microbial metabolites that can compete with drug metabolism [55]. Therefore, the gut microbiota influences drug-related liver injury (Figure 2).

It is estimated that the human gastrointestinal tract sheds 1011 epithelial cells per day [56]. Adult stem cells in the intestinal crypts are capable of maintaining a continuous epithelial cell population, which is essential for barrier function. From the apical to the basal membranes, tight junctions, adhesion junctions, bridging junctions, and gap junctions are formed by various proteins and molecules between adjacent epithelial cells, which together strengthen intercellular junctions and form information channels, among which tight junctions are the most important. The tight junctions of intestinal epithelial cells are located at the junction between the apical and basolateral plasma membrane domains and consist of transmembrane proteins, signaling molecules, and membrane-associated scaffolding proteins. Transmembrane proteins include tight junction-associated marvel proteins (TAMPS), Claudin, and junctional adhesion molecules (JAMs), which bind scaffolding proteins such as zonula occludens 1 (ZO1), ZO2, and ZO3, thereby linking transmembrane proteins to the actin cytoskeleton [57]. The intestinal epithelium is supported by a thick layer of mucus containing mucins (MUCs), which are highly glycosylated glycoproteins produced mainly by goblet cells [58]. In the colon, transmembrane MUCs and secreted MUCs are part of a dual system containing an inner dense layer containing a small number of microorganisms and an outer sparse layer [59]. The intestine contains many immune cells, type-I interferon-producing plasmacytoid dendritic cells, innate lymphoid cells, mucosa-associated invariant T cells, and γδ T cells [60]. The immune system contributes to the stability of the intestinal barrier by secreting IgA and antimicrobial peptides. Secreted IgA is produced by the lamina propria plasma cells [61]. IgA is secreted as a dimer and can facilitate the crosslinking and entrapment of bacteria, limiting the settlement and growth of potential pathogens. Symbiotic microorganisms such as *Bacteroides* fragilis use IgA cross-linking to promote colonization [62,63]. In addition to binding the pathogen itself, secreted IgA neutralizes the secreted bacterial toxin [64]. Antimicrobial peptides include lysozyme, α-defensins and β-defensins, C-type lectins, and cathelicidins, which have antimicrobial activity and are secreted by Paneth cells located at the base of the intestinal crypts. Due to the diversity of antimicrobial peptides and their ability to target bacterial membranes, most bacteria do not develop resistance to these proteins [65].

The gut microbiota promotes health in part by enhancing the intestinal barrier through direct and indirect mechanisms. The gut microbiota prevents pathogens from invading the intestinal ecosystem by competing for space and nutrients, which is called colonization resistance [66,67]. Apollo Stacy et al. [68] also found that intestinal pathogenic bacterial infections “train” the host to increase bile acid metabolism and its product taurine in the intestine, which is catalyzed to sulfide by bacteria enriched with taurine, thereby inhibiting the respiration of pathogenic bacteria and enhancing colonization resistance of the flora. However, damage to the intestinal barrier increases intestinal permeability, leading to the entry of PAMPs into the bloodstream and activation of the innate immune response. The liver and the intestines are connected by a portal circulation. In this system, blood flows from the intestine through the portal vein, then into the hepatic vein, and then back to the heart and lungs. Therefore, PAMPs in portal blood are first encountered by immune cells in the liver. PAMPs, such as bacteria, LPS, and viral RNAs, activate pathogen recognition receptors, such as TLR4, on Kupffer cells and other immune cells to induce innate immune responses. Subsequently, hepatic inflammation contributes to liver injury and disease progression [69,70].

Although the gut has an impact on the liver, through the portal circulation, liver metabolites and others are also released back into the gut through hepatic bile flow and mediators. Thus, the liver and gut microbiota interact with each other. For example, environmental factors affecting liver function (age, sex, diet, toxins, etc.) also influence gut physiology and gut microbial diversity [4,67,71].

## 4. Effects of Gut Microbiota on DILI

### 4.1. Effects of Gut Microbiota on APAP-Induced Liver Injury

APAP, one of the most commonly used antipyretic and analgesic drugs, has been in widespread use worldwide since 1955. It is found in a variety of preparations and is widely used both as a single-ingredient drug and as an ingredient in many combinations of over-the-counter and prescription medications [72]. Generally, APAP is a safe drug when used in therapeutic doses (1–4 g/day) for the treatment of fever and pain, while overdose may lead to hepatotoxicity and ALF [44,73]. It is responsible for approximately 50% of ALF cases in the United States and some European countries [74,75] and is the most common single cause of ALF. In recent years, numerous studies have shown that intestinal-level events such as dysbiosis, intestinal barrier dysfunction, and intestinal inflammation play an important role in APAP-induced liver injury. The gut microbiota is associated with susceptibility to APAP-induced hepatotoxicity [6,76]. The metabolism of APAP by enteric epithelial cells in vitro attenuates hepatocellular toxicity, but gut inflammation amplifies the induced hepatotoxicity [77].

#### 4.1.1. Effects of Gut Microbiota Abundance and Diversity on APAP-Induced Liver Injury

Drug metabolism has been thought to occur primarily through the liver. However, studies have shown that orally administered xenobiotics are absorbed by the gut microbiota prior to absorption into the bloodstream [78,79]. The pharmacokinetic parameters of orally administered APAP may also be influenced by fluctuations in the gut microbiota. In rodents, for example, the urinary excretion of APAP and its metabolites in them was significantly reduced after treatment with antibiotics [79,80]. The sulfation of phenolic compounds (e.g., cresol, acetaminophen, and tyrosine) can be catabolized by acyltransferases from intestinal bacteria such as Eubacterium rectale A-44 or Enterobacter amnigenus AR [81].

Changes in the diversity and abundance of the gut microbiota may also be involved in attenuating APAP hepatotoxicity. *Akkermansia muciniphila* is a strictly anaerobic Gram-negative species belonging to Verrucomicrobia, which constitutes 0.5~5% of the human intestinal microflora [82]. Mitochondrial oxidative stress and dysfunction are considered the main pathogenic mechanisms of APAP hepatotoxicity, while inflammation is regarded as a necessary exacerbating factor during the progression of APAP-induced liver injury (AILI) [83]. *Akkermansia muciniphila* significantly reduces APAP-induced oxidative stress and the inflammatory response, effectively attenuating AILI. It restores the balance of reduced glutathione/oxidized glutathione, enhances superoxide dismutase activity, and reduces pro-inflammatory cytokines, macrophage infiltration, as well as neutrophil infiltration. Additionally, *Akkermansia muciniphila* maintains intestinal barrier function, reshapes the damaged microbiota, and promotes the secretion of SCFAs [84]. *Bacteroides* vulgatus acts as a probiotic in the body, inhibiting the colonization of pathogenic microorganisms and alleviating oxidative stress and liver injury caused by APAP [85,86].

The most common means of changing the composition of the gut microbiota is to take probiotics orally, which can maintain the integrity of the intestinal barrier, reduce the production of toxic substances, and improve liver function. It has been found in mice that the intake of probiotics may cause fluctuations in the absorption of oral drugs by interfering with intestinal microbiome-mediated drug metabolism [87]. The subsequent effect on microbiota metabolism may lead to altered systemic concentrations of intact drugs. Thus, co-administration of probiotics with drugs may result in changes in the pharmacokinetic parameters of the drug. It was shown that the probiotics Enterococcus lactis *IITRHR1* and *Lactobacillus acidophilus MTCC447* attenuated APAP-induced hepatotoxicity by modulating the antioxidant capacity of the liver and the expression of key apoptotic/anti-apoptotic proteins [88]. MegaSporeBiotic^TM^ probiotic capsules are composed of a probiotic blend of spores from five *Bacillus* species, ameliorating histopathological liver injury and reducing pro-inflammatory cytokine levels, which suggests a protective effect of probiotic *Bacillus* spores against APAP-induced acute liver injury [89].

However, it has also been shown that changes in the diversity and abundance of the gut microbiota are factors that exacerbate APAP hepatotoxicity. Vancomycin was found to alter the composition of the gut microbiota in mice, reducing the abundance of Gram-positive bacteria in the gut and increasing the levels of 2-hydroxybutyric acid in the cecum and serum. Ultimately, it increased glutathione (GSH) levels in the liver and ameliorated APAP-induced liver injury in mice [90]. The hepatotoxic phenotype of germ-free mice treated under the same conditions was less than that of conventionally fed mice. The extent of liver damage in germ-free mice within 8 h after paracetamol intoxication was comparable to that of conventionally housed animals. However, there was no significant trend toward lower serum bilirubin and creatinine levels in germ-free mice, so they may have less liver damage at later time points [91]. Therefore, it becomes possible to treat patients with DILI using intestinal cleansing antibiotics as a therapeutic intervention to modify the gut microbiota. P-cresol has such potential. Sulfotransferase in hepatocytes converts p-cresol absorbed into the liver into p-cresol sulfate, and acetaminophen is also a substrate for sulfotransferase, so p-cresol reduces the ability of sulfotransferase to convert APAP. Endogenous p-cresol is mainly produced by intestinal microorganisms such as *Clostridium difficile*. Individual differences in the abundance of intestinal microorganisms such as *Clostridium difficile* lead to individual differences in p-cresol content, which may contribute to the different susceptibilities of individuals to APAP hepatotoxicity [92,93].

In summary, the diversity and abundance of gut microbiota affect APAP hepatotoxicity and become a potential new target for therapeutic interventions for DILI through antibiotics and probiotics.

#### 4.1.2. Effects of Gut Microbial Metabolites on APAP-Induced Liver Injury

Gut microbial metabolites are key factors in regulating the host response to drugs. The gut microbiota modulates susceptibility to liver disease by producing several bioactive products with multiple physiological or pathological functions. For example, 5-methoxyindoleacetic acid secreted from *Lactobacillus rhamnosus GG* protects mice against APAP- and ethanol-induced hepatotoxicity by activating the Nrf2 protein [94]. The gut microbiota and its metabolites are involved in the regulation of oxidative stress and inflammation, playing key roles in drug-induced hepatotoxicity [6,95].

Liver injury due to APAP has a significant daily variation, and it is more severe when the drug is administered at night than in the morning [96]. The intestinal microbial metabolite 1-phenyl-1,2-propanedione (PPD) was at least partially involved in the daily changes in hepatotoxicity induced by APAP through the reduction in GSH levels [6]. Some strains, such as *Enterococcus faecalis*, *Clostridium difficile*, *Citrobacter freundii*, and *E. coli*, generate PPD. PPD synergistically enhanced APAP-induced liver injury both in vivo and in vitro. PPD can deplete GSH, and when APAP is consumed in excess, N-acetyl-p-benzoquinone imine competes with the intestinal flora metabolite PPD for GSH, resulting in GSH deficiency and a large accumulation of N-acetyl-p-benzoquinone imine, which in turn triggers severe and diurnal variation in acute liver injury [6].

SCFAs are fermentation products produced by the gut microbiota and consist of fatty acids with 2 to 6 carbon atoms. A total of 74 gut bacteria have been found to produce SCFAs, the majority of them belonging to the well-known probiotic genera *Lactobacillus*, *Bifidobacterium*, and *Clostridium* [97]. SCFAs not only provide energy for intestinal epithelial cells but also have a significant impact on intestinal proliferation, differentiation, and function, contributing to the maintenance of the intestinal barrier and regulation of host metabolism [98]. Recent studies have shown a link between SCFAs and AILI [84]. *Akkermansia muciniphila* alleviates APAP-induced liver injury by enhancing the production of SCFAs [84,99]. Furthermore, ampicillin exacerbates APAP-induced liver injury by reducing butyrate levels, which can be reversed by supplementation with Lactobacilli [99]. Butyrate is a nutrient for intestinal cells that promotes cell regeneration, maintains intestinal barrier function, and possesses anti-inflammatory properties [100].

Recent research has shown that the compound phenylpropionic acid produced by gut microbiota can also help mitigate APAP-induced liver injury [76]. The authors systematically compared the sensitivity of C6BL/6 mice with different gut microbiota but similar genetics from two suppliers, Jackson and Taconic, to APAP-induced hepatotoxicity and identified a gut bacterial metabolite called phenylpropionic acid [76]. Cytochrome P450 2E1 is the major enzyme responsible for converting acetaminophen into its toxic metabolite [101]. Supplementation of phenylpropionic acid reduced the liver cytochrome P450 2E1 levels and alleviated APAP-induced hepatotoxicity in mice, while also reducing liver damage induced by carbon tetrachloride mediated by cytochrome P450 2E1 [76].

Some gut microbiota also have protective effects on the liver through their metabolites, such as *Clostridium* sporogenes, whose metabolite indole derivatives can inhibit the expression of pro-inflammatory genes and enhance the expression of anti-inflammatory genes in the liver. The enzymatic activity of *Lactobacillus vaginalis β-galactosidase* in releasing daidzein inhibits Fdps-mediated ferroptosis in hepatocytes, thereby ameliorating APAP-induced liver injury in mice [102]. Ferroptosis may be the cause of APAP-induced liver injury [103,104]. In a mouse model of APAP-induced liver failure, ferroptosis inhibitors effectively suppressed mouse mortality caused by excessive APAP [105].

In summary, part of the mechanism by which the gut microbiota regulates drug-related liver injury is through its metabolites, and the gut microbiota can produce a large number of metabolites, many of which are metabolized by the liver. Some metabolites might enhance APAP hepatotoxicity, while some metabolites attenuate APAP hepatotoxicity.

#### 4.1.3. Effects of Gut Barrier on APAP-Induced Liver Injury

The gut and liver are anatomically linked by the biliary tract, portal system, and circulating products from the immune system, and the gut microbiota forms the “gut-liver axis”. Microorganism-associated molecular patterns and PAMPs from the intestine reach the liver through the portal system and enter the body circulation through the mesenteric lymph nodes, where they bind to TLRs and lead to activation of inflammatory pathways. The gut microbiota and the intestinal barrier are interconnected to achieve a complex network of interactions that, under physiological conditions, are in balance and contribute to the dynamic homeostasis and health of the body [7,8,9]. Intestinal barrier dysfunction and dysbiosis can lead to the development of diseases in the liver and other organs. Failure of any aspect of this barrier can lead to the translocation of microorganisms into the bloodstream and result in a sustained inflammatory response. Mengwei et al. found that intestinal barrier disruption may be associated with APAP-induced hepatotoxicity and that CCL7-mediated impairment of the intestinal barrier integrity may be an important factor in APAP-induced hepatotoxicity. The authors suggested that maintaining intestinal integrity may be a novel strategy for counteracting APAP-induced liver injury [106]. Impaired gut barrier integrity is an extrahepatic characteristic of APAP intoxication, enhancing bacterial translocation and exacerbating liver inflammation [107]. Propionic acid and butyric acid are also negatively associated with intestinal barrier dysfunction, thereby restoring the damaged intestinal barrier and improving oxidative stress and inflammation [84]. APAP triggers significant changes in the composition of the microbiota and subsequently negatively affects the intestinal barrier integrity [31]. Loss of intestinal barrier integrity leads to increased intestinal permeability, which promotes systemic bacterial translocation and the entry of large amounts of harmful substances into the liver, thereby exacerbating the inflammatory response and hepatotoxicity of APAP. In summary, APAP can lead to the destruction of the intestinal barrier, and a damaged intestinal barrier can aggravate APAP-induced liver injury; thus, protecting the intestinal barrier might protect against APAP-induced liver injury.

### 4.2. Effect of Gut Microbiota on the Hepatotoxicity of Other Drugs

#### 4.2.1. Effect of Gut Microbiota on the Anti-Tuberculosis Drug-Induced Liver Injury

Tuberculosis (TB) is one of the most common causes of death from infectious disease in adults worldwide and has been considered a global public health emergency for the past 25 years [108]. Isoniazid (INH), rifampin (RIF), pyrazinamide (PZA), and ethambutol (EMB) are the four drugs in the first-line antimicrobial regimen used clinically to treat drug-susceptible TB. In recent years, numerous articles have shown that treatment with anti-tuberculosis drugs leads to significant changes in the number of gut microbiota. The number of operational taxonomic units (OTUs), Shannon’s index, and Pielou’s evenness index were significantly reduced, indicating a substantial reduction in microbial diversity after treatment with these four drugs for two weeks [109]. A significant reduction in the diversity of both intestinal bacterial and fungal flora in patients was also found on first-time anti-tuberculosis drugs, and the altered intestinal bacterial microbiota was mainly associated with the order *Clostridium* [110]. *Clostridium* plays a vital role in gut microbiota homeostasis and immune balance, as it can significantly improve liver steatosis, repair damaged liver cells, and significantly reduce ALT, AST and ALP levels, which has a protective effect on the liver [111]. This may be a major contributing factor to DILI. The results suggested that the impact of gut microbiota on isoniazid-induced liver injury was associated with the immune response, and the difference in INH-DILI sensitivity was related to the structure of the gut microbiota [112]. Changes in the structure of the gut microbiota by continuous exposure to INH resulted in the tolerance to liver injury, and probiotics such as *Bifidobacterium* might play an important role in INH-DILI and its tolerance phenomenon.

Additionally, anti-tuberculosis drugs cause changes in the beneficial commensal bacteria in the intestinal flora. It was found that INH treatment led to a reduction in the intestinal flora of mice like *Bacteroides*, *Campylobacter*, and *Lactobacillus* genus, which had shown significant beneficial immunomodulatory features [113]. At the same time, *Akkermansia muciniphila* are also known for its probiotic potential and illustrate the key function of intestinal barrier protection and systemic inflammation inhibition [114,115]. It was also confirmed that the abundance of *Akkermansia muciniphila* bacterium was restricted and decreased during liver injury in rats caused by anti-tuberculosis drugs, while the levels of mRNA and protein of tumor necrosis factor alpha and interleukin 6 were reduced in rats supplemented with *A. muc. bacterium*. More specifically, *Akkermansia muciniphila* supplementation may alleviate anti-tuberculosis drug-induced liver injury by improving the mechanical and immune barriers of the rat intestinal mucosa, reducing LPS levels, and thereby effectively alleviating liver inflammatory factor levels [116]. Meanwhile, most of the genera with increased abundance belonged to *Ruminococcaceae* and *Lachnospiraceae* in the phylum Thick-walled Bacteria, which have the ability of secondary bile acid production. Secondary bile acids promote liver tumorigenesis by inhibiting natural killer T-cell activation through suppression of the CXC chemokine ligand 16 protein [117]. In other words, the intestinal flora can affect the disease process by regulating the production of secondary bile acids that modulate immune cells in the liver.

Probiotics can alleviate liver damage caused by anti-tuberculosis drugs. *Lactobacillus casei* is commonly found in the intestines of mammals [118]. The administration of anti-tuberculosis drugs results in the activation of the TLR4-NF-κB-MyD88 pathway, leading to liver injury. However, when high doses of *Lactobacillus casei* are administered, the activation of NF-κB and MyD88 proteins is inhibited [119]. Consequently, this reduces oxidative stress and inflammation, ultimately alleviating liver injury. Anti-tuberculosis drugs impaired the intestinal barrier in mice, reduced gut microflora diversity, and altered its composition. *Lactobacillus casei* strengthened the intestinal barrier and restored gut microflora to near-normal composition [119]. In conclusion, probiotics can alleviate anti-tuberculosis DILI and restore the imbalance of gut microecology.

#### 4.2.2. Effect of Gut Microbiota on Iron-Induced Liver Injury

The iron homeostasis of the organism and the intestinal flora also have a mutually regulated relationship. After dietary iron enters the digestive tract, most of it enters the colon, except for a small portion that is absorbed by the small intestine. Since iron is a key factor for the growth and reproduction of most bacteria, the iron content in the intestine can influence the abundance and diversity of the intestinal flora, which further regulates the metabolic or immune response of the host. Additional iron supplementation given to children aged 6–14 years found reduced numbers of *Lactobacillus* in the intestinal bacteria and a mild inflammatory response in an examination six months later [120]. However, the supplementation of iron in infants approximately 6 months of age adversely affected the gut microbiota, leading to increased levels of intestinal pathogens and inflammatory responses and an increased chance of diarrhea [121]. Excess iron greatly increased lipid peroxidation in the duodenal mucosa but only mildly increased protein oxidation and that excess iron caused severe swelling of mitochondria and slight swelling of the endoplasmic reticulum, resulting in oxidative stress and expression of inflammatory factors, which contributed to the apoptosis of hepatocytes [34]. At the same time, a decrease in intestinal flora leads to increased intestinal permeability and impaired barrier function, which increases the transport of iron, harmful bacteria, and endotoxins into the circulation, and systemic inflammation accompanied by oxidative stress can lead to further iron deposition in the liver. In this vicious cycle, liver injury will continue to worsen. In summary, excess iron not only acts directly on the liver to cause liver injury but also affects the intestinal flora, destroys the intestinal barrier, affects intestinal osmotic pressure, and weakens intestinal protection, contributing to the formation of DILI.

#### 4.2.3. Effect of Gut Microbiota on the Herbal Medicine-Induced Liver Injury

Recently, more attention has been given to the interactions between herbal medicines and the gut microbiota. The interaction between herbal medicines and the gut microbiota is bidirectional: on the one hand, the intestinal flora will metabolize herbal medicines, which may increase efficacy, reduce toxicity, or may generate toxic metabolites. On the other hand, herbal medicines can regulate the structure and metabolic function of the flora by selectively inhibiting or promoting the growth of different types of intestinal microorganisms.

Relevant studies have proved that the gut microbiota can produce low polarity and relatively stable molecular masses of herbal medicine metabolites through hydrolysis, oxidation, reduction, and isomerization reactions, which can accelerate intestinal absorption and improve the bioavailability of herbal medicine, thus affecting the efficacy and toxicity of herbal medicine [122]. It has been reported that the gut microbiota is involved in the metabolism of triptolide. The gut microbiota may exert its detoxification effect by metabolizing triptolide to nontoxic metabolites. Preincubation with antibiotics to eliminate gut microbiota could enhance the activation of the NLR family pyrin domain containing 3 (NLRP3) inflammasome in C57BL/6 mouse livers and aggravate the inflammation and hepatotoxicity induced by triptolide [123]. Similarly, it was found that gut microbiota-derived propionate could protect the liver against inflammatory injury in C57BL/6 mice, and the hepatotoxicity induced by triptolide could be reversed by gut microbial transplantation [124]. Oral amygdinoside is hydrolyzed by β-glucosidase of the intestinal flora to produce the toxic substance hydrocyanic acid, which triggers a serious toxic reaction [125]. Aconitine Chinese medicines such as Chuanwu and Caowu have been confirmed to be converted into mono- and di-lipids with lower toxicity under the acylation and esterification of intestinal microorganisms [126].

Most glycosides in the Huangqin decoction can be digested and absorbed by the body through the catalytic deglycosylation of gut microbiota [127]. Ginsenosides can be transformed into hydrophobic compounds under the combined action of gastric juice and intestinal microorganisms, such as protopanaxadiol-type ginsenosides, which are mainly converted into compounds K and ginsenoside Rh2. Compared with protopanaxadiol-type ginsenosides, the transformed metabolite compound K exhibits more effective pharmacological effects such as antitumor, anti-inflammatory, antidiabetic, anti-allergic, and neuroprotective effects [128]. Chinese herbs, such as Huanglian detoxification soup, can directly affect the microbiota that produces SCFAs in the intestine [129].

The use of herbal medicines can directly affect the composition of the gut microbiota. In mice with cholestatic liver injury, fed with the Huangqin decoction (Astragali Radix and Glycyrrhizae Radix et Rhizoma), it was found that the *Prevotellaceae_NK3B31_group*, Alistipes closely related to the inflammatory response, and *Gordonibacter (ODU383)* were significantly decreased in abundance and the expression of inflammatory factors such as NLRP3 was reduced [117,130]. This result further suggests that the preventative effect of the Huangqi decoction on liver inflammation and liver injury may occur through affecting the abundance and species of intestinal flora, thereby reversing intestinal ecological dysregulation and refining aspects of barrier integrity dysfunction. In contrast, differences were found when studying the effects of total licorice saponin and aqueous extract on the intestinal flora of liver-injured rats. They found that compared with the Glycyrrhiza aqueous extract group, the abundance of flora of family *S24-7* and *Lactobacillus* spp. in the total saponin group was significantly increased, while the abundance of flora of *Trichoderma* spp. and *Clostridium_sensu_stricto_1* were significantly decreased. The total saponin group also had an increased relative proportion of intestinal probiotics, especially the proportion of *Lactobacillus* spp. The experimental results showed that both total licorice saponin and the aqueous extract of licorice could improve chronic liver injury and exert different modulatory effects on the intestinal flora of liver-injured rats [131]. However, overdose administration of herbal medicines may cause liver injury. Taken together, gut microbiota possesses an effect on herbal medicine hepatotoxicity, and measures should be taken to maintain the balance of gut microbiota when using herbal medicines in the clinic. However, the relationship between herbal medicine and gut microbiota needs to be further explored, and detailed mechanisms will provide guidelines in the clinic.

## 5. Future Directions

Over the past decade, a burgeoning body of evidence has elucidated the intricate symbiosis between the gut microbiota and hepatic function, implicating this complex interplay in the pathogenesis of drug-induced liver injury (DILI). This insight has unveiled novel therapeutic avenues, including fecal microbiota transplantation, targeted antibiotic therapy, and the administration of probiotics, prebiotics, and synbiotics. Strategically modulating the gut microbiome to suppress the generation of hepatotoxic metabolites may alleviate hepatic stress and confer hepatoprotection. Conversely, enhancing the proliferation of beneficial commensals can foster the production of salutary gut-derived metabolites, thereby imparting a hepatoprotective effect. Despite the promising therapeutic implications underscored by both clinical and experimental research, the field is still nascent, with a paucity of clinical evaluation tools and a limited understanding of the microbiota–host dynamics. Nonetheless, it is anticipated that through meticulous and judicious investigation, bolstered by the rapid evolution of modern analytical technologies, research into the gut microbiome will revolutionize the management of DILI. This could encompass more precise risk stratification, improved therapeutic interventions, and the realization of considerable economic and societal dividends. The prospect of harnessing the gut microbiota in the realm of DILI therapeutics is not only scientifically intriguing but also holds a transformative potential for patient care. 

## Data Availability

Data are available within the article.
